# Supplementary material for: Integrative multi-omics reveals a regulatory and exhausted T-cell landscape in CLL and identifies galectin-9 as an immunotherapy target
Source: Nat Commun. 2025 Aug 7;16:7271. doi: 10.1038/s41467-025-61822-x (PMC12331977; doi:10.1038/s41467-025-61822-x)
Supplement: Supplementary file 1 — Supplementary Information [file 41467_2025_61822_MOESM1_ESM.pdf]

Supplementary Figure 1

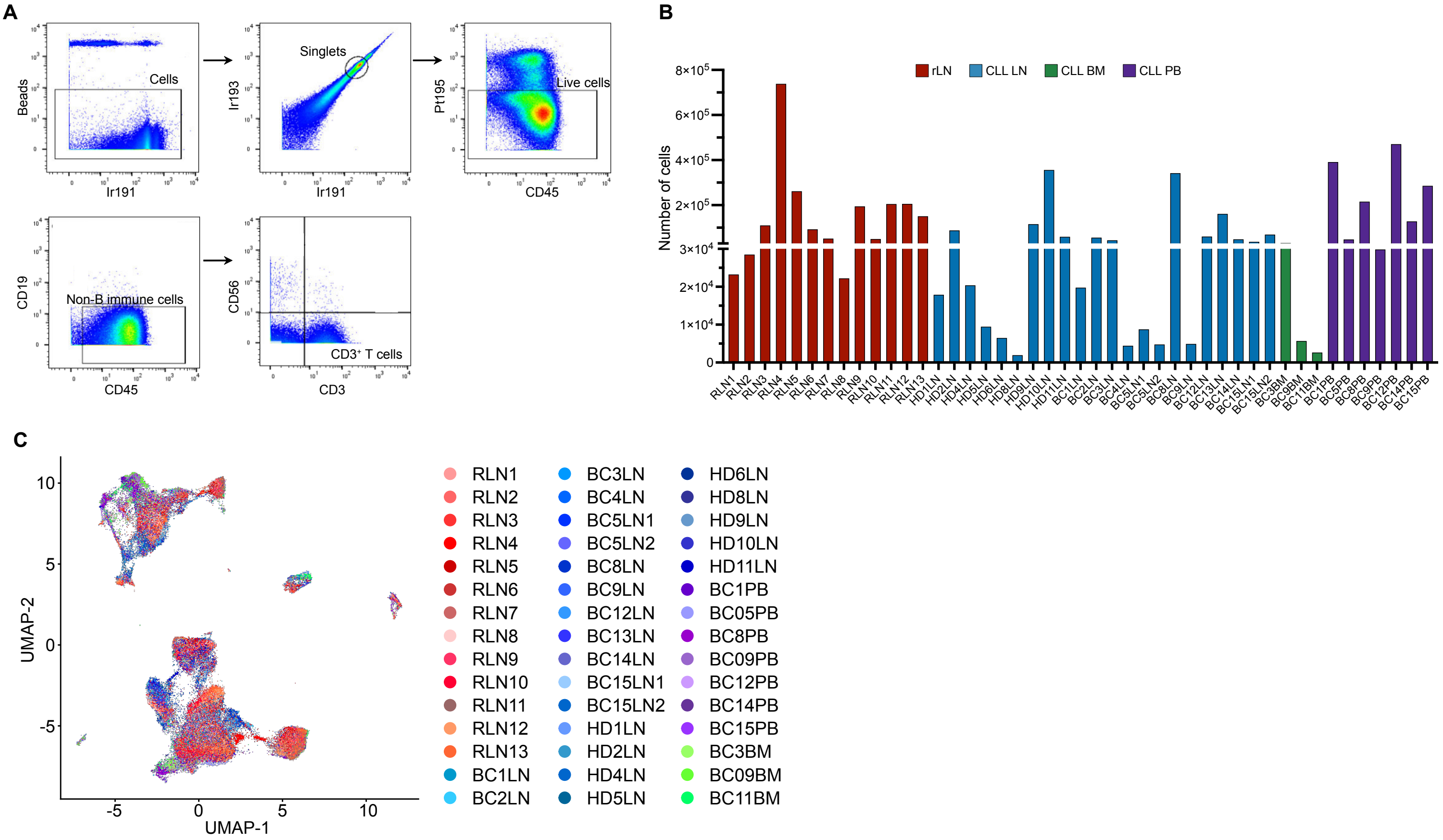

Supplementary Figure 1 (related to Figure 1): Quality control of mass cytometry data to define the T-cell landscape in CLL tissues at the single-cell resolution

A) Gating strategy used to select live, $CD45^+$ $CD3^+$ T cells: gates were placed on cells (Beads vs. Ir191), singlets (Ir193 vs. Ir191) and live cells ($Pt195^-$), which were gated and exported as .fcs files. $CD3^+$ cells were further analyzed. **B)** Number of T cells per analyzed sample in reactive lymph nodes (rLN), CLL lymph nodes (LN), bone marrow (BM), and peripheral blood (PB). **C)** UMAP plot depicting sample distribution. Source data are provided as a Source Data file FigS1.

Supplementary Figure 2

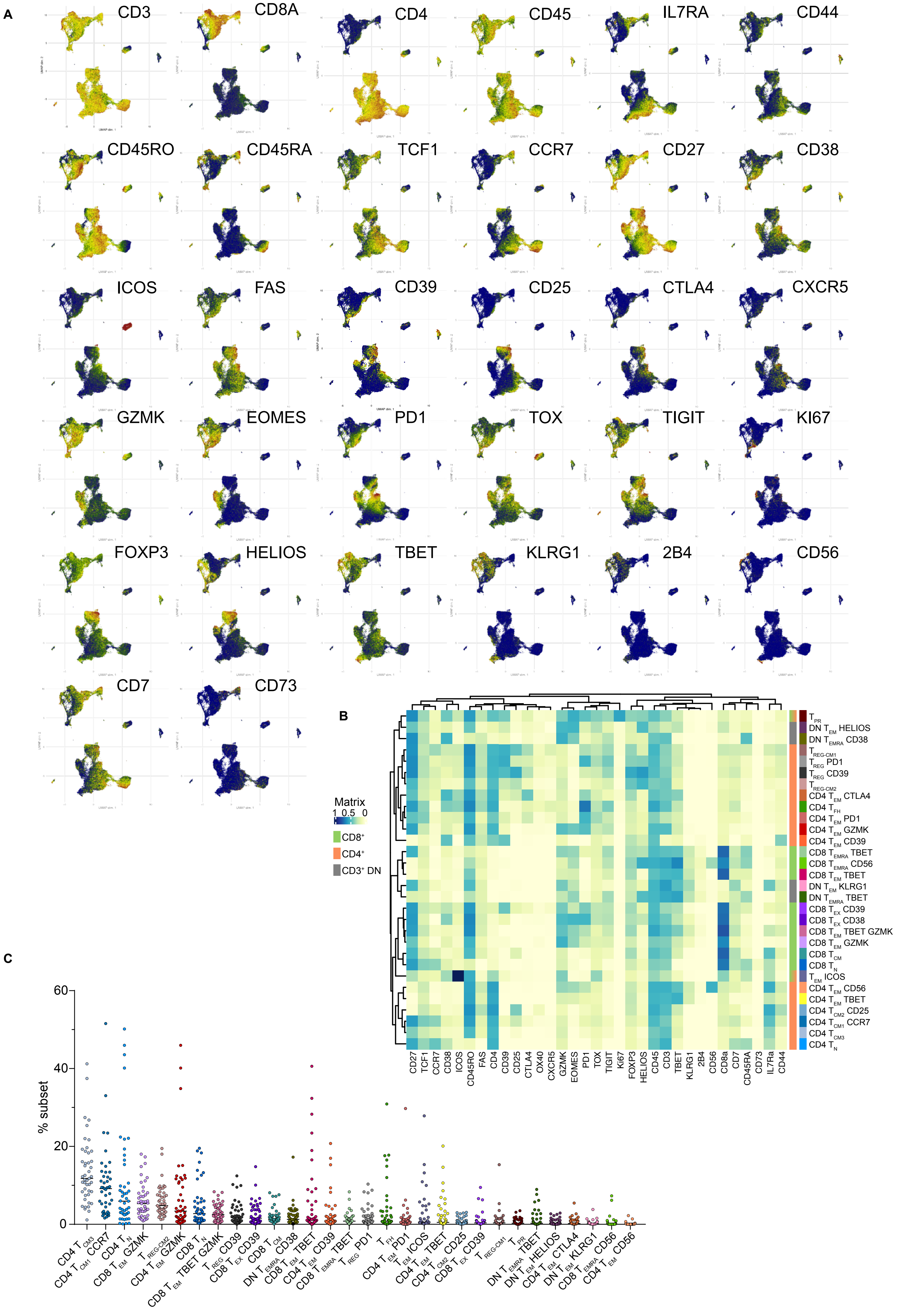

Supplementary Figure 2 (related to Figure 1): Overview of all protein markers analyzed by mass cytometry and frequencies of identified T-cell subsets

A) Projection of all protein markers analyzed by mass cytometry. Cells are colored based on the normalized protein expression. **B)** Heatmap showing median protein expression of the 33 markers considered (columns) for each cluster (rows) identified. Both markers and clusters were grouped based on hierarchical clustering. **C)** Cell subset frequency for each cluster. Each dot represents one sample. Clusters are ordered from highest to lowest median frequency. Source data are provided as a Source Data file FigS2.

Supplementary Figure 3

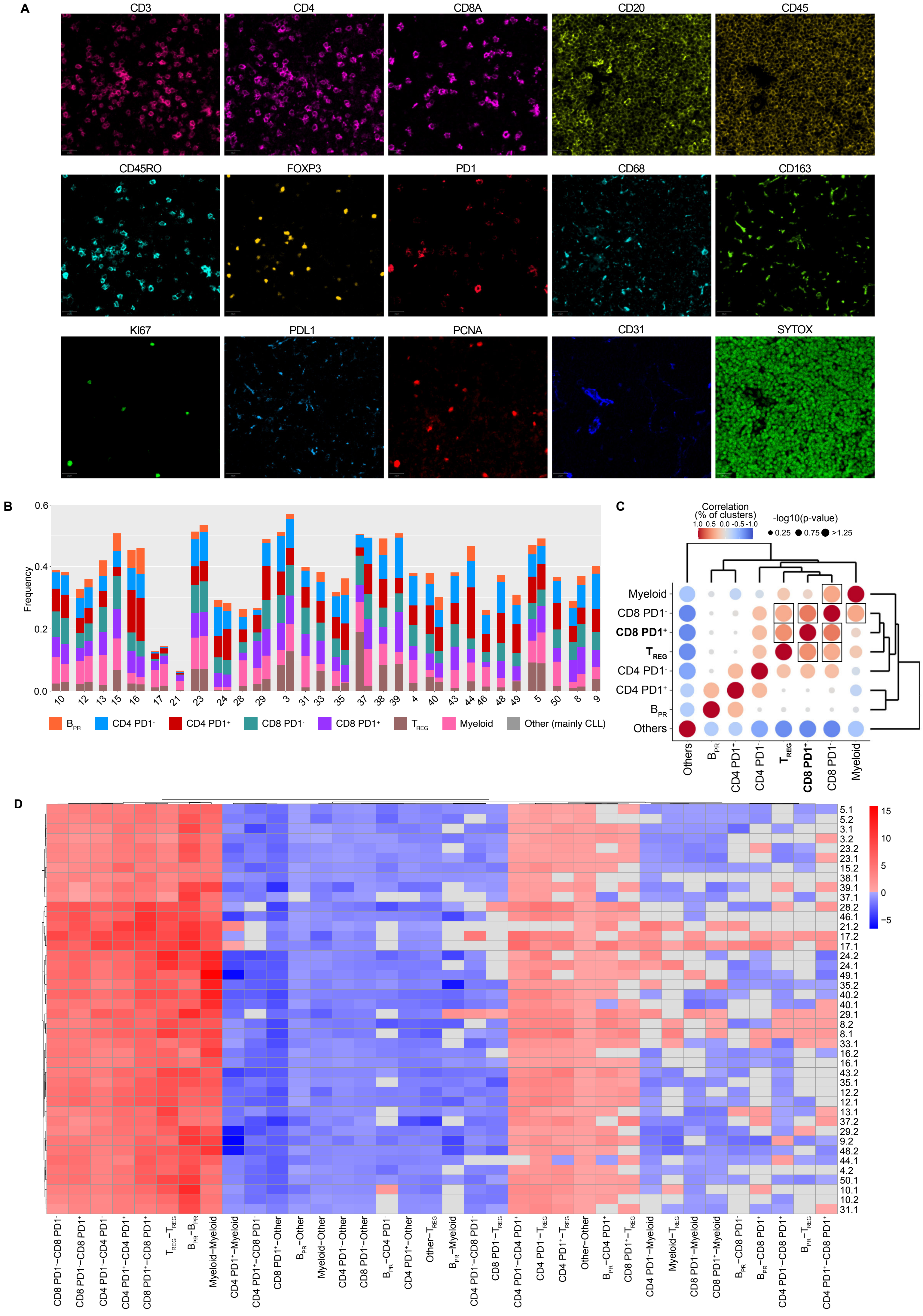

Supplementary Figure 3 (related to Figure 2): Overview of multiplex staining of CLL LNs and frequencies and correlations of identified cell types

A) Representative images of 15-color multiplex staining of tissue cores of CLL LNs. **B)** Frequency of all cell types identified using the 15-color multiplex staining. Colors represent the individual cell types, and each core is represented by the individual column – with cores from the same patient grouped together - in the bar plot (n = 42). “Other (mainly CLL)” cells make up the rest of the cells and are not depicted. **C)** Heatmap showing the Pearson correlation coefficient and its associated p-value of the multiplex staining cell subset proportions from the 42 CLL LN cores, corresponding to the worst performing subset of all leave-one-out patient samples. The correlation for each cell subset pair was then defined as the result among subsets for which the p-value was highest. Significant results are depicted with black borders. **D)** Heatmap showing the significance score of pairwise interacting cell types (columns) for each core (rows) (n=42) as generated by Giotto. Cell type pairs and cores were both grouped based on hierarchical clustering. Source data are provided as a Source Data file FigS3.

Supplementary Figure 4

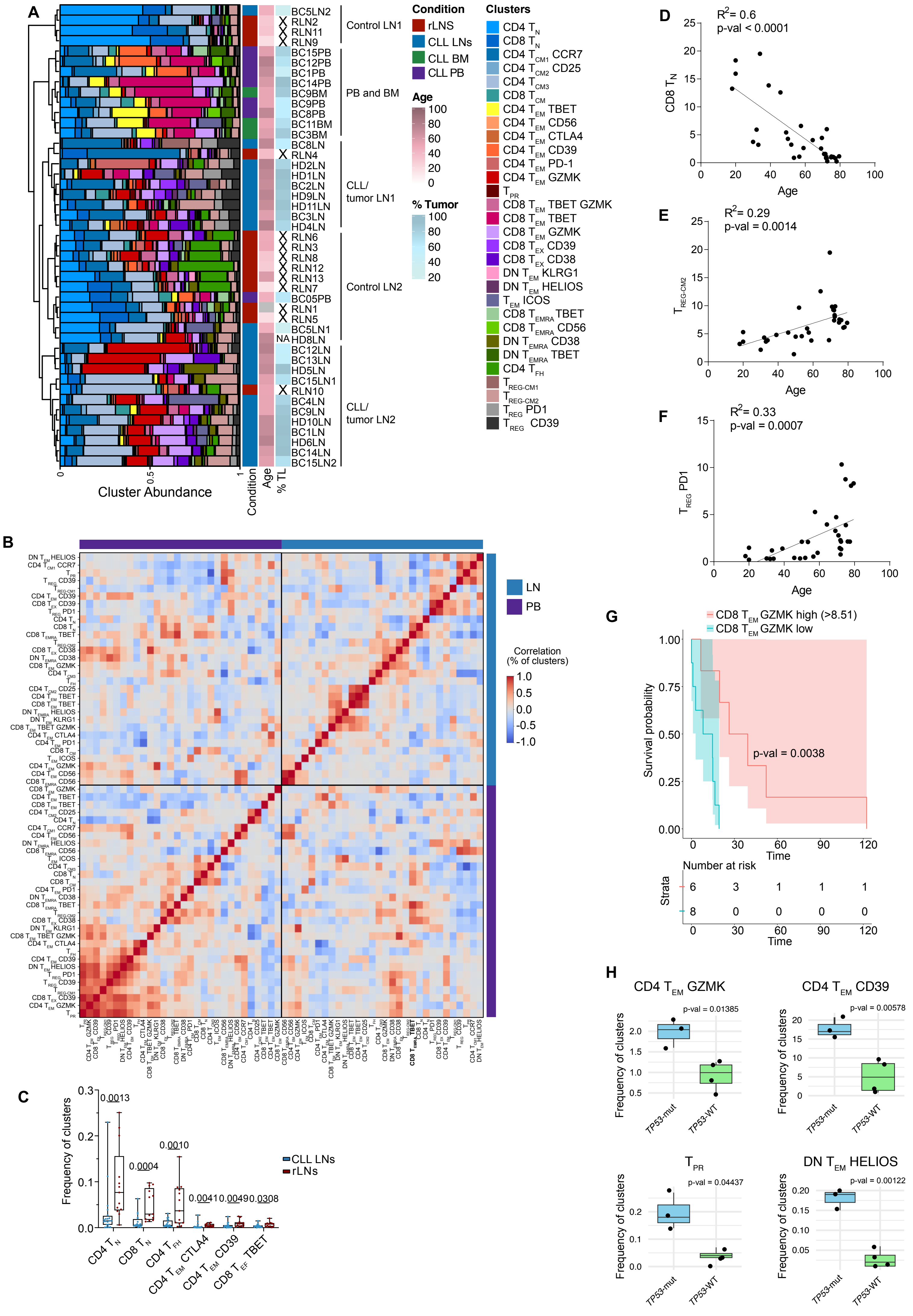

Supplementary Figure 4 (related to Figure 3): T-cell composition in all samples analyzed by mass cytometry and correlations of T-cell subsets and clinical data

A) Hierarchical clustering (with Pearson correlation and complete linkage) of samples based on the T-cell subset abundances in each sample. Cell subsets are color-coded as defined in Figure 1B. Row annotations indicate sample condition (BM, bone marrow, LN, lymph node, and PB, peripheral blood), age (years), and tumor load (TL). **B)** Heatmap showing the Pearson correlation coefficient and its associated p-value of cell subset proportions from the 7-paired CLL LN and PB samples, corresponding to the worst-performing subset of all leave-one-out patient samples. The correlation for each cell subset pair was then defined as the result among subsets for which the p-value was highest. **C)** Boxplot showing cell subset abundances out of total T cells in CLL LNs (n=20) and rLNs (n = 13). **D-F)** Pearson correlation plot between age and frequency of CD8 T_{N} cells (C), $T_{REG\ CM2}$ cells (D), and T_{REG} PD1 cells (E). Each symbol represents an individual patient sample. **G)** Survival probability in CLL patients with high or low frequencies of CD8 T_{EM} GZMK cells (n = 14). **H)** Frequency of CD4 T_{EM} GZMK, CD4 T_{EM} CD39, T_{PR} and DN T_{EM} HELIOS subsets out of total T cells in *TP53*-mutated (mut; n = 3) versus unmutated (WT; n = 4) CLL patients. Boxplots represent the 25th to 75th percentiles with the median as the central line, whiskers indicate minimal and maximal value. Statistical significance was tested by two-sided Wilcoxon rank sum test (C and H). Source data are provided as a Source Data file FigS4.

Supplementary Figure 5

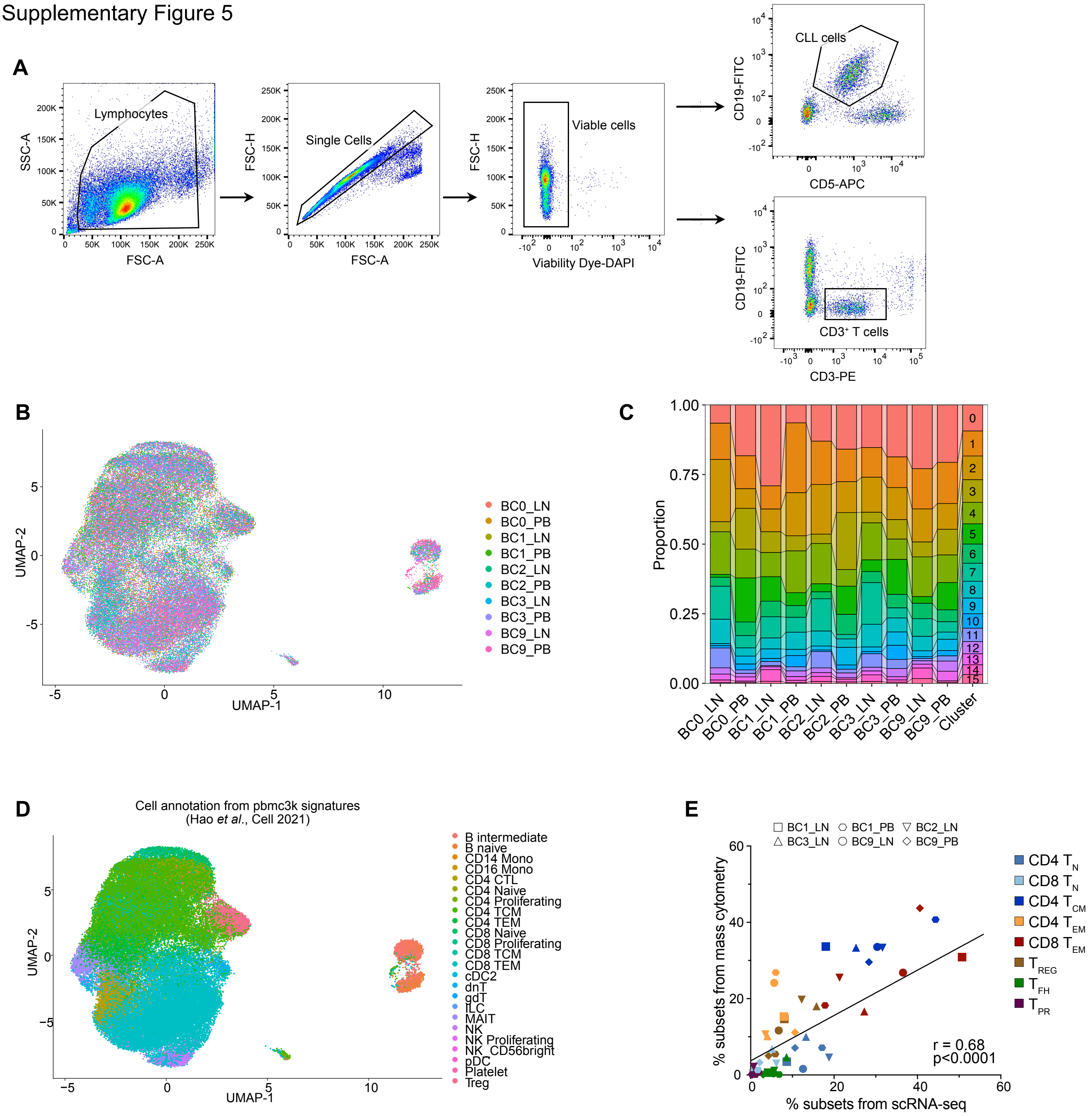

Supplementary Figure 5 (related to Figure 4): Quality control of single-cell RNA-seq data of CLL PB and LN samples and correlation with mass cytometry results

A) Gating strategy used to isolate live, $CD45^+$ $CD3^+$ T cells and CLL cells from PB and LN samples of CLL patients: gates were placed on cells (SSC-A vs FSC-A), singlets (FSC-H vs FSC-A) and live cells (Negative for viability dye). $CD45^+$ cells were further gated for CLL cells ($CD19^+$ $CD5^+$) or T cells ($CD19^-$ $CD3^+$). **B)** UMAP plot depicting sample distribution. **C)** Subset distribution per sample. Colors correspond to the clusters defined in Figure 4A. **D**) UMAP colored according to PBMC3K data set[21] applied to the data set of 5 CLL LNs and 5 CLL PB samples. **E)** Pearson correlation of major T-cell subset frequencies determined with scRNA-seq and the mass cytometry data for the 5 CLL patient samples analyzed with both techniques. Source data are provided as a Source Data file FigS5.

Supplementary Figure 6

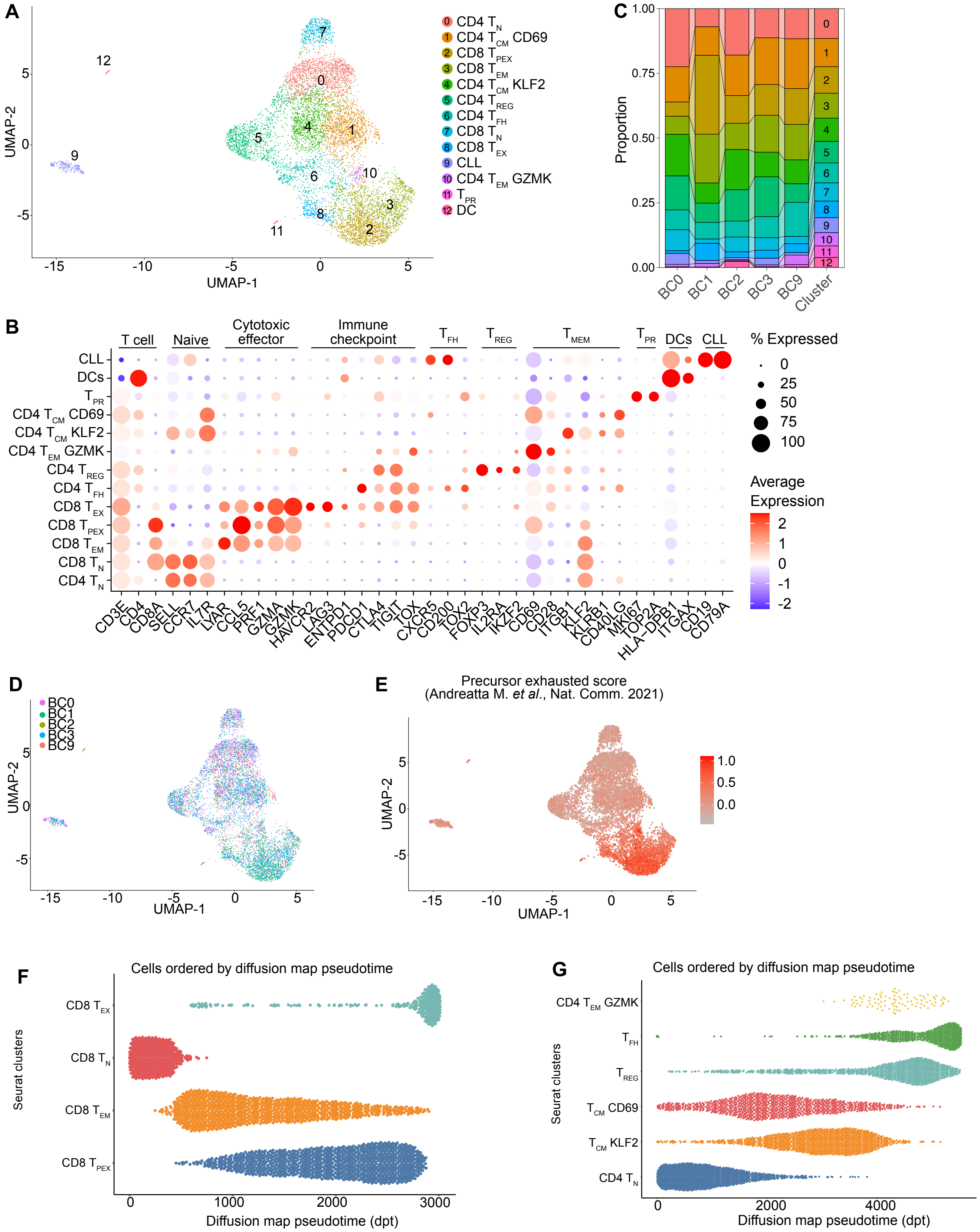

Supplementary Figure 6 (related to Figure 4): Cluster annotations and analyses of single-cell RNA-seq data of CLL LN samples

A) UMAP plot of 8,576 cells from 5 CLL LN samples identifying 13 clusters, including T cells, dendritic cells (DC) and CLL cells. **B)** Dot plot of the expression of marker genes in the 13 cell clusters. **C)** Subset distribution per sample. Colors correspond to the clusters defined in A. **D)** UMAP plot colored by sample. **E)** UMAP plot colored according to the average expression of the precursor exhaustion gene signature from Andreatta *et* al[24]. **F-G)** $CD8^+$ (F) and $CD4^+$ (G) T-cell subsets ordered by diffusion pseudotime. Source data are provided as a Source Data file FigS6.

Supplementary Figure 7

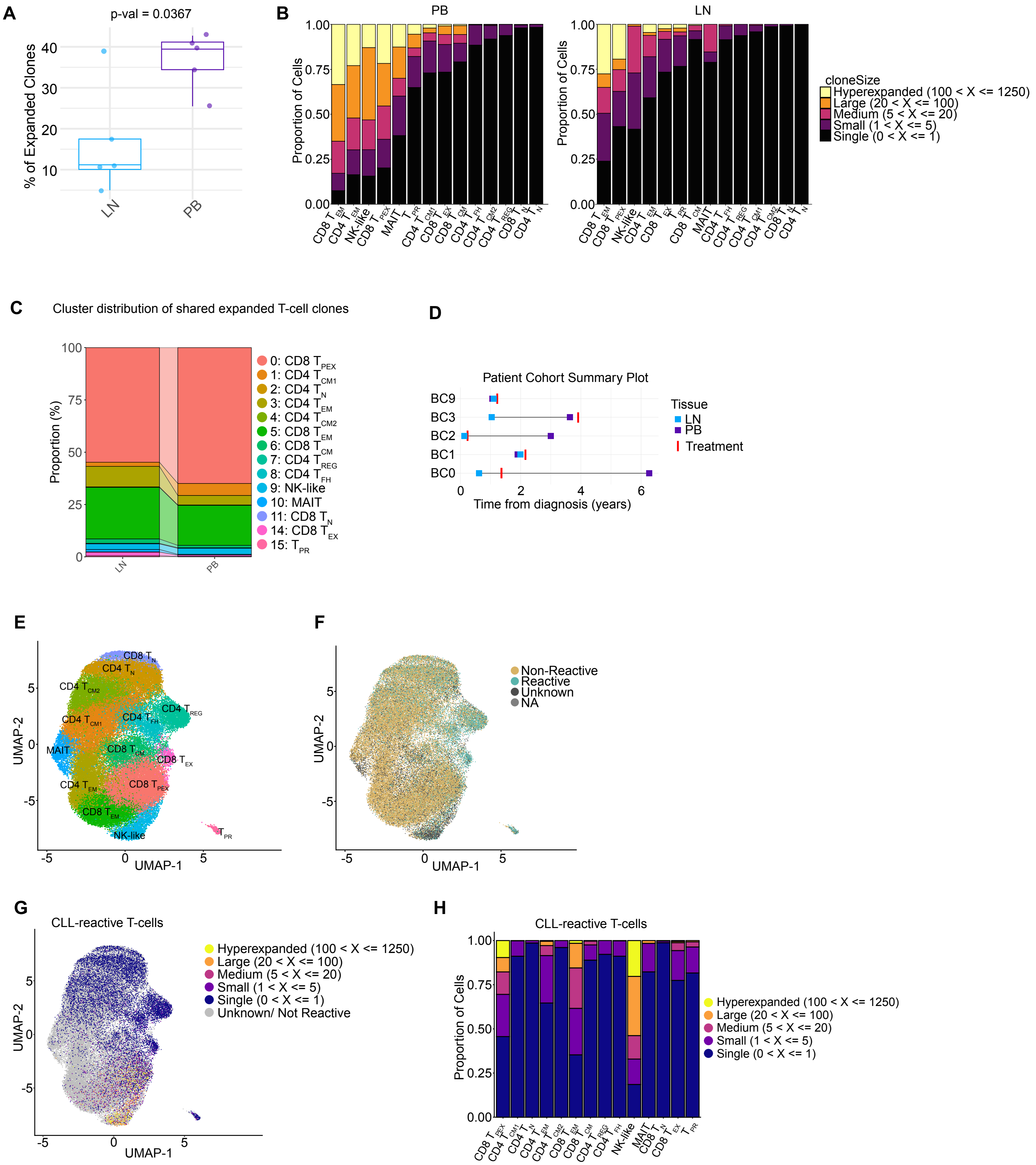

Supplementary Figure 7 (related to Figure 5): TCR analyses of CLL PB and LN samples to define clonal expansion and predict CLL-reactivity of T-cell subsets

A) Percentage of clonally expanded T cells (clonotypes of size >1) in LN (n = 5) vs PB (n = 5). Boxplots represent the 25th to 75th percentiles with the median as the central line, whiskers indicate minimal and maximal value. Each dot represents a sample. Statistical significance was tested by two-sided Wilcoxon rank sum test. **B)** Percentage of T-cell clone sizes based on TCR-seq data. **C)** Cluster distribution of shared expanded T-cell clones between LN and PB. **D)** Patient cohort summary displaying time (years) of sampling (LN and PB) and treatment after diagnosis. **E)** UMAP plot colored according to the clusters identified. **F)** UMAP plot colored according to tumor-reactivity as predicted by predicTCR. NA: no TCR information available. **G)** UMAP with tumor-reactive cells colored according to the clone size. Tumor-reactive cells are displayed on top for a better visualization. **H)** Proportion of T-cell clone sizes of CLL-reactive T cells predicted by predicTCR.

Supplementary Figure 8

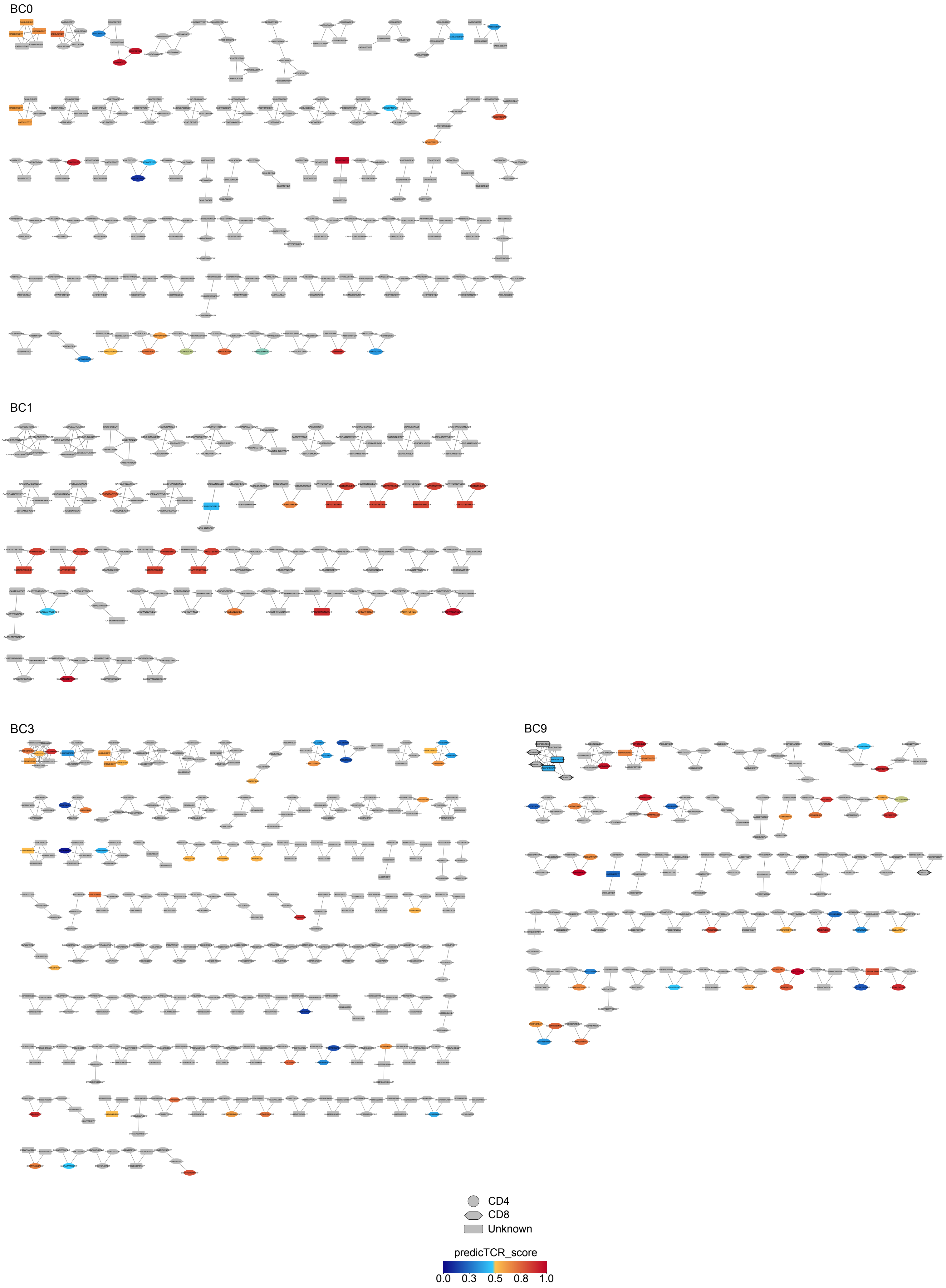

Supplementary Figure 8 (related to Figure 5): GLIPH2 analysis of TCR data of CLL PB and LN samples

Clusters of convergently recombined TCRs of samples BC0, BC1, BC3, and BC9 identified by GLIPH2 containing multiple CLL T-cell-derived TCRs predicted to be tumor-reactive (orange-red), as well as TCRs found in LN or PB samples for which no scSEQ data and predicTCR scores were available (grey). TCRs for which CD4/CD8 status could not be determined due to lack of scSEQ data are illustrated as rectangular nodes.

Supplementary Figure 9

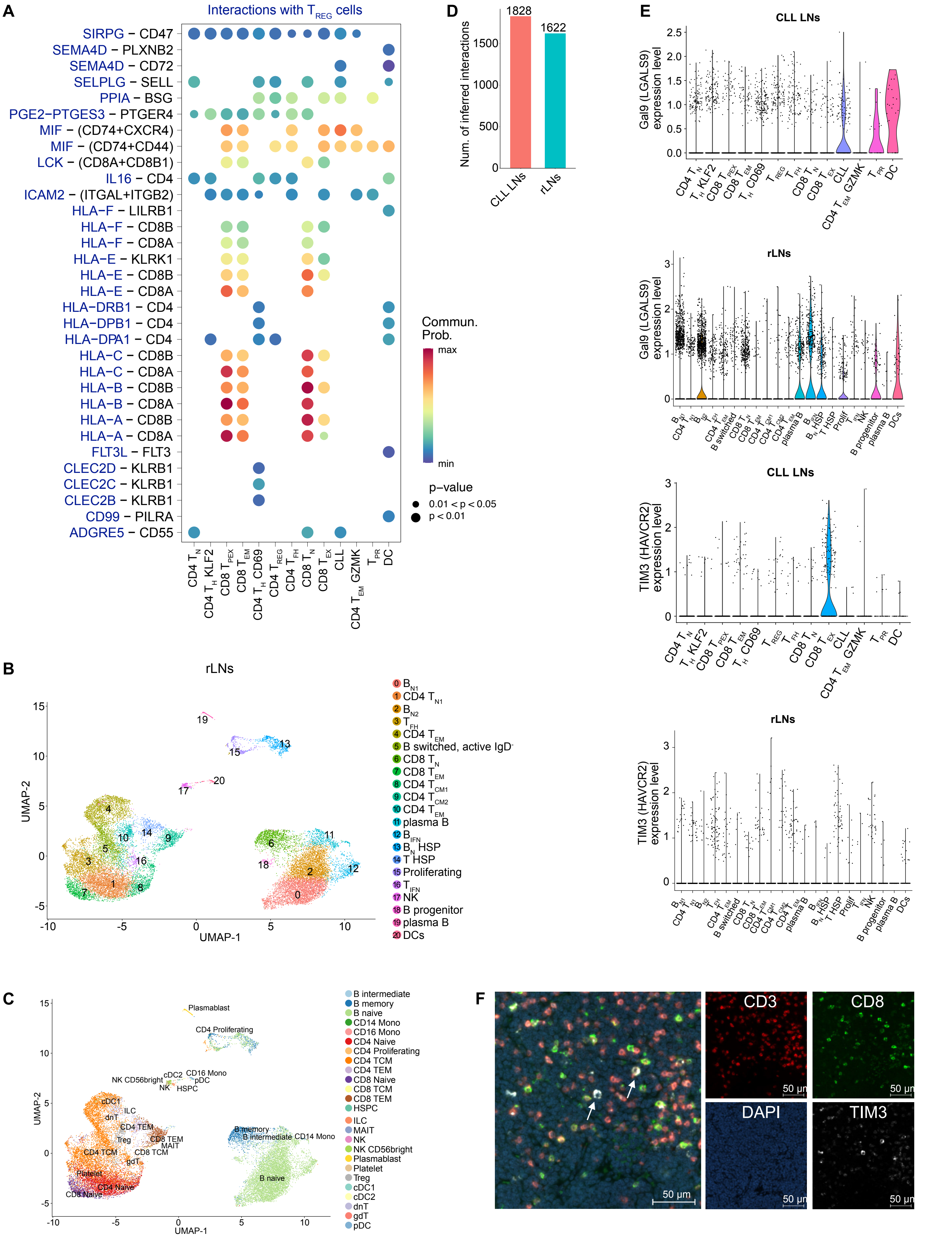

Supplementary Figure 9 (related to Figure 6): Interactome analysis using CellChat and validation of *LGALS9* and TIM3 expression in CLL LNs

A) Cell-cell communication network using CellChat was analyzed on scRNAseq data from 5 CLL LNs. Heatmap plot depicting the list of significant ligand-receptor pairs between CD4 T_{REG} cells (molecule in blue) and all the other cell subsets (molecule in black). The dot color and size represent the calculated communication probability and p-values of differential communication, respectively. **B)** UMAP plot of 20,673 cells from 5 rLNs analyzed by scRNA-seq from Aoki *et* al.[34] identifying 21 clusters, including 9 T-cell clusters, 8 B-cell clusters, proliferating cells, NK cells, and DCs. **C)** UMAP plot where PBMC3K data set[21] was mapped to the 5 rLNs data set from Aoki *et* al.[34]. **D)** Differential cell-cell communication networks between CLL LNs and rLNs was analyzed using CellChat. The plot displays the number of inferred interactions between T cells and B cells in rLN samples, or T cells and CLL cells in CLL LN samples. **E)** Violin plots showing the expression distribution of *LGALS9* (top two plots) and *HAVCR2* (bottom plots) genes in the CLL LN data set and the rLN data set from Aoki *et* al.[34]. Each dot represents one cell. **F)** Representative microscopy images showing CD3, CD8, TIM3 and DAPI as single stains and overlayed image (left) in a CLL LN tissue section. Arrows indicate $CD3^+$ $CD8^+$ $TIM3^+$ cells. Source data are provided as a Source Data file FigS9.

Supplementary Figure 10

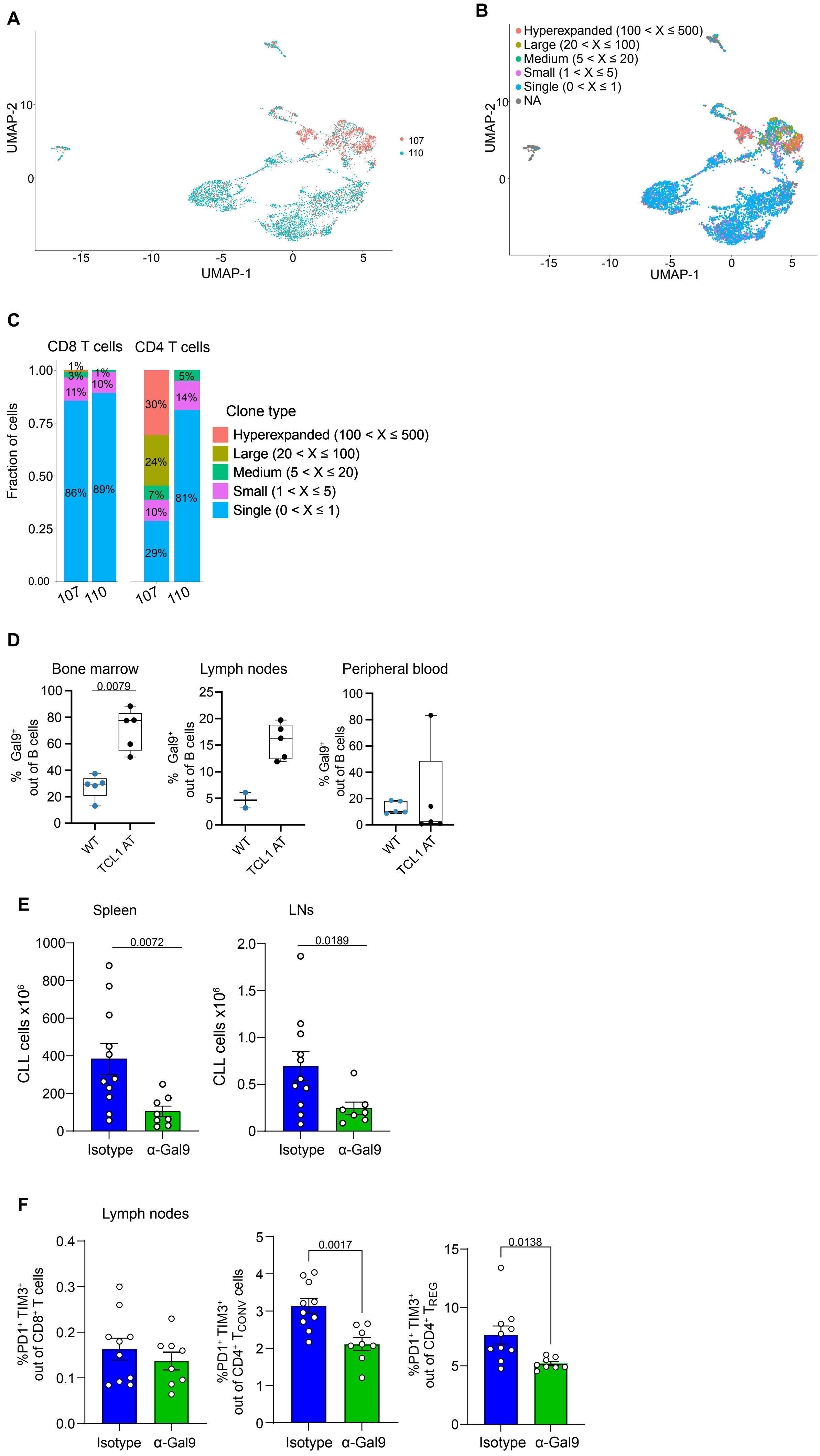

Supplementary Figure 10 (related to Figure 7): Single-cell RNA- and TCR-seq data of T cells from CLL mouse model and evaluation of galectin-9 as therapy target

A-C) Two spleen samples of mice after adoptive transfer of TCL1 leukemia (TCL1 AT) were analyzed by scRNA-seq. **A)** UMAP plot depicting sample distribution of scRNA-seq data of splenic T cells from 2 TCL1 AT mice. **B)** UMAP plot colored according to the T-cell clone size based on the TCR-seq data. NA: no TCR information available. **C)** Bar plot indicating the percentage (rounded values are indicated) of single, small, medium, large and hyperexpanded sized clones in $CD8^+$ (left) and $CD4^+$ (right) T cells for the two mouse samples analyzed**. D)** Percentage of galectin-9^+ ($Gal9^+$) B cells and CLL cells from bone marrow, lymph nodes and peripheral blood of wild-type control (WT; n = 5) and TCL1 AT (n = 5) mice. Boxplots represent the 25th to 75th percentiles with the median as the central line, whiskers indicate minimal and maximal value. Each symbol represents an individual mouse. Statistical significance was tested by two-sided Wilcoxon rank sum test. **E)** Absolute number of $CD19^+$ $CD5^+$ CLL cells in spleen (right) and lymph nodes (left) of isotype antibody- (n = 11) and anti-galectin-9 (α-Gal9)-treated (n = 8) mice. **F)** Percentage of $PD1^+$ $TIM3^+$ cells out of $CD8^+$, $CD4^+$ T_{CONV}, and $CD4^+$ T_{REG} cells in lymph nodes of isotype antibody- (n = 11) and α-Gal9-treated (n = 8) mice. Each symbol represents an individual mouse, and statistical significance was tested by unpaired t-test with Welch approximation. Bars indicate mean ± SEM. Only significant p-values are shown. Source data are provided as a Source Data file FigS10.

Supplementary Figure 11

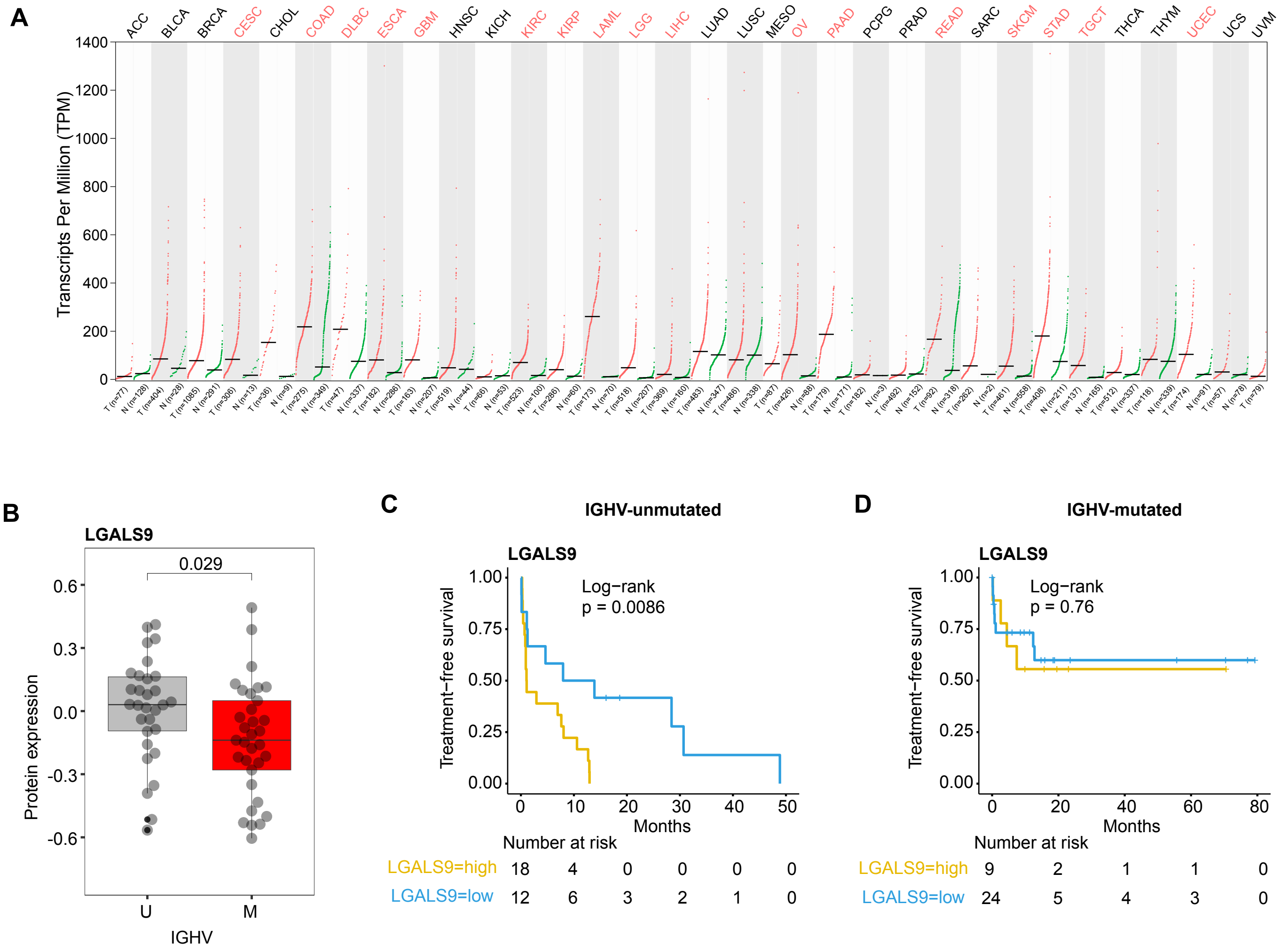

Supplementary Figure 11 (related to Figure 8): Differential expression of *LGALS9* in tumor versus healthy tissue and in *IGHV*-mutated versus -unmutated CLL including prognostic relevance

A) Comparative analysis of *LGALS9* transcript levels in tumor versus healthy tissue in ACC (Adrenocortical Carcinoma), BLCA (Bladder Urothelial Carcinoma), BRCA (Breast invasive carcinoma) CESC (Cervical squamous cell carcinoma and endocervical adenocarcinoma), CHOL (Cholangiocarcinoma), COAD (Colon adenocarcinoma), DLBC (Lymphoid Neoplasm Diffuse Large B-cell Lymphoma), ESCA (Esophageal carcinoma), GBM (Glioblastoma multiforme), HNSC (Head and Neck squamous cell carcinoma), KICH (Kidney Chromophobe), KIRC (Kidney renal clear cell carcinoma), KIRP (Kidney renal papillary cell carcinoma), LAML (Acute Myeloid Leukemia), LGG (Brain Lower Grade Glioma), LIHC (Liver hepatocellular carcinoma), LUAD (Lung adenocarcinoma), LUSC (Lung squamous cell carcinoma), MESO (Mesothelioma), OV (Ovarian serous cystadenocarcinoma), PAAD (Pancreatic adenocarcinoma), PCPG (Pheochromocytoma and Paraganglioma), PRAD (Prostate adenocarcinoma), READ (Rectum adenocarcinoma), SARC (Sarcoma), SKCM (Skin Cutaneous Melanoma), STAD (Stomach adenocarcinoma), TGCT (Testicular Germ Cell Tumors), THCA (Thyroid carcinoma), THYM (Thymoma), UCEC (Uterine Corpus Endometrial Carcinoma), UCS (Uterine Carcinosarcoma) and UVM (Uveal Melanoma) using the standard processing pipeline GEPIA2 with default cut-off settings[49]. Tumors with differential expression of *LGALS9* are indicated in red. Statistical differences were assessed by limma model with adjusted p-values (Benjamini and Hochberg FDR). Each dot represents a patient or healthy donor sample. **B)** Galectin-9 protein levels in *IGHV*-unmutated (U; n = 30) versus *IGHV*-mutated (M; n = 33) CLL patients. Each dot represents a patient. Statistical significance was assessed by two-sided Wilcoxon signed-rank test. **C-D)** Time-to-treatment in C) *IGHV*-unmutated (n = 30) and D) *IGHV*-mutated (n = 33) CLL patients with high or low galectin-9 protein levels[50]. Statistical differences were assessed by Cox regression model. Source data are provided as a Source Data file FigS11.

Supplemental Data Titles

Suppl. Data 1: Information on patients and donors

Suppl. Data 2: Antibodies, reagents and software

Suppl. Data 3: Cell cluster annotations

Suppl. Data 4: Correlations with clinical data

Suppl. Data 5: Single-cell RNA-seq summary and statistical data from T_{EX} and T_{PEX} signatures

Suppl. Data 6: HLA-typing and VDJdb results of TCR-seq data
