# Supplementary material for: Integrative multi-omics reveals a regulatory and exhausted T-cell landscape in CLL and identifies galectin-9 as an immunotherapy target
Source: Nat Commun. 2025 Aug 7;16:7271. doi: 10.1038/s41467-025-61822-x (PMC12331977; doi:10.1038/s41467-025-61822-x)
Supplement: Supplementary file 2 — Description of Additional Supplementary Files [file 41467_2025_61822_MOESM2_ESM.pdf]

Description of Additional Supplementary Files

Supplementary Data 1: Information on patients and donors

Supplementary Data 2: Antibodies, reagents and software

Supplementary Data 3: Cell cluster annotations

Supplementary Data 4: Correlations with clinical data

Supplementary Data 5: Single-cell RNA-seq summary and statistical data from T_{EX} and T_{PEX} signatures

Supplementary Data 6: HLA-typing and VDJdb results of TCR-seq data
