# Supplementary material for: Integrative multi-omics reveals a regulatory and exhausted T-cell landscape in CLL and identifies galectin-9 as an immunotherapy target
Source: Nat Commun. 2025 Aug 7;16:7271. doi: 10.1038/s41467-025-61822-x (PMC12331977; doi:10.1038/s41467-025-61822-x)
Supplement: Supplementary file 9 — Reporting Summary [file 41467_2025_61822_MOESM9_ESM.pdf]

nature portfolio

Corresponding author(s): Martina Seiffert

Last updated by author(s): Jul 2, 2025

Reporting Summary

Nature Portfolio wishes to improve the reproducibility of the work that we publish. This form provides structure for consistency and transparency in reporting. For further information on Nature Portfolio policies, see our Editorial Policies and the Editorial Policy Checklist.

Statistics

For all statistical analyses, confirm that the following items are present in the figure legend, table legend, main text, or Methods section.

n/a	Confirmed	
[ ]	[x]	The exact sample size (*n*) for each experimental group/condition, given as a discrete number and unit of measurement
[ ]	[x]	A statement on whether measurements were taken from distinct samples or whether the same sample was measured repeatedly
[ ]	[x]	The statistical test(s) used AND whether they are one- or two-sided<br>*Only common tests should be described solely by name; describe more complex techniques in the Methods section.*
[ ]	[x]	A description of all covariates tested
[ ]	[x]	A description of any assumptions or corrections, such as tests of normality and adjustment for multiple comparisons
[ ]	[x]	A full description of the statistical parameters including central tendency (e.g. means) or other basic estimates (e.g. regression coefficient) AND variation (e.g. standard deviation) or associated estimates of uncertainty (e.g. confidence intervals)
[ ]	[x]	For null hypothesis testing, the test statistic (e.g. *F*, *t*, *r*) with confidence intervals, effect sizes, degrees of freedom and *P* value noted<br>*Give P values as exact values whenever suitable.*
[x]	[ ]	For Bayesian analysis, information on the choice of priors and Markov chain Monte Carlo settings
[x]	[ ]	For hierarchical and complex designs, identification of the appropriate level for tests and full reporting of outcomes
[ ]	[x]	Estimates of effect sizes (e.g. Cohen's *d*, Pearson's *r*), indicating how they were calculated

Our web collection on statistics for biologists contains articles on many of the points above.

Software and code

Policy information about availability of computer code

Data collection	Details are provided in the Methods section
Data analysis	Details are provided in the Methods section.<br>Code availability<br>All code related with CyTOF and scRNA-seq analyses is available on GitHub (https://github.com/mzapatka/Tcell_CLL).

For manuscripts utilizing custom algorithms or software that are central to the research but not yet described in published literature, software must be made available to editors and reviewers. We strongly encourage code deposition in a community repository (e.g. GitHub). See the Nature Portfolio guidelines for submitting code & software for further information.

Data

Policy information about availability of data

All manuscripts must include a data availability statement. This statement should provide the following information, where applicable:

- Accession codes, unique identifiers, or web links for publicly available datasets
- A description of any restrictions on data availability
- For clinical datasets or third party data, please ensure that the statement adheres to our policy

Data availability
The human single-cell RNA- and TCR-sequencing data including matrix files generated in this study have been deposited in the European Genome-phenome Archive

(EGA) under the accession ID EGAS00001006864 (https://ega-archive.org/studies/EGAS00001006864). The sequencing raw data are available under restricted access, which can be obtained by request to the Data Access Committee. Mouse single-cell RNA- and TCR-sequencing data are available on Gene Expression Omnibus under the accession ID GSE221395.
https://www.ncbi.nlm.nih.gov/geo/query/acc.cgi?acc=GSE221395.
The CyTOF data used in this study are available in the Zenodo database under the DOI: 10.5281/zenodo.15606759. https://doi.org/10.5281/zenodo.15606759
Source data are provided with this paper.

Research involving human participants, their data, or biological material

Policy information about studies with human participants or human data. See also policy information about sex, gender (identity/presentation), and sexual orientation and race, ethnicity and racism.

Reporting on sex and gender	Deatails are provided in the extensive Methods section and in Suppl. Data 1 and 4 describing the sex of the individuals and correlations with clinical data.
Reporting on race, ethnicity, or other socially relevant groupings	Information on race, ethnicity or other socially relevant groupings is not provided as the cohort size was too small to allow for any groupings based on such data.
Population characteristics	Details are provided in Suppl. Data 1 and 4.
Recruitment	Patient selection was based on availability of rare samples. Inclusion of all cases with paired blood and tissue samples. No compensation was provided for patients and healthy donors for participating in this study.
Ethics oversight	The Ethics Committees that approved the study are provided in the Ethics declaration of the manuscript.<br>Samples were included after informed consent and according to the guidelines of the Ethics Committees of the medical faculties of the University of Barcelona and the University of Heidelberg.

Note that full information on the approval of the study protocol must also be provided in the manuscript.

Field-specific reporting

Please select the one below that is the best fit for your research. If you are not sure, read the appropriate sections before making your selection.

[x] Life sciences [ ] Behavioural & social sciences [ ] Ecological, evolutionary & environmental sciences

For a reference copy of the document with all sections, see nature.com/documents/nr-reporting-summary-flat.pdf

Life sciences study design

All studies must disclose on these points even when the disclosure is negative.

Sample size	Sample size was based on availability of rare samples and on the throughput size of the used technologies. Inclusion of all cases with paired blood and tissue samples was prioritized.
Data exclusions	Exclusion criteria for scRNAseq, CyTOF and image analyses are provided in the extensive Methods section.
Replication	Description of reproducibility of results in independent experiments is provided in the manuscript.
Randomization	Randomization of mice in treatment groups was performed based on tumor load.
Blinding	Blinding of treatment groups for the preclinical study in mice was performed.

Reporting for specific materials, systems and methods

We require information from authors about some types of materials, experimental systems and methods used in many studies. Here, indicate whether each material, system or method listed is relevant to your study. If you are not sure if a list item applies to your research, read the appropriate section before selecting a response.

Materials & experimental systems

n/a	Involved in the study	
	x	Antibodies
x		Eukaryotic cell lines
x		Palaeontology and archaeology
	x	Animals and other organisms
	x	Clinical data
x		Dual use research of concern
x		Plants

Methods

n/a	Involved in the study	
x		ChIP-seq
	x	Flow cytometry
x		MRI-based neuroimaging

Antibodies

Antibodies used	The list of antibodies is provided in Suppl. Data 2.
Validation	Only commercially available and validated antibodies were used in this manuscript.

Animals and other research organisms

Policy information about studies involving animals; ARRIVE guidelines recommended for reporting animal research, and Sex and Gender in Research

Laboratory animals	A detailed description on the mouse strains used in this study is provided in the Methods section.<br>Female C57BL/6 wild-type (WT) mice were purchased from Charles River Laboratories (Germany) or Janvier Labs (France).
Wild animals	No wild animals were used.
Reporting on sex	Animal experiments were performed with female mice. The use of female mice was justified due to a more homogeneous disease development in those compared to male mice, and due to no major differences observed in the T-cell landscape of male and female CLL patients.
Field-collected samples	No field-collected samples were used.
Ethics oversight	The organizations that approved the animal experiments are indicated in the Ethics declararations of the manuscript.<br>All experiments involving laboratory animals were conducted in pathogen-free animal facilities at the German Cancer Research Center in Heidelberg or the Luxembourg Institute of Health in Luxembourg with the approval of the Regierungspräsidium Karlsruhe (G-77/19 and G-112/21) and the Luxembourg Ministry for Agriculture (#LUPA 2019/21), respectively.

Note that full information on the approval of the study protocol must also be provided in the manuscript.

Clinical data

Policy information about clinical studies

All manuscripts should comply with the ICMJEguidelines for publication of clinical research and a completedCONSORT checklist must be included with all submissions.

Clinical trial registration	Patient samples were not collected as part of a clinical trial.
Study protocol	*Note where the full trial protocol can be accessed OR if not available, explain why.*
Data collection	Clinical data were collected at the University hospitals in Heidelberg and Barcelona.
Outcomes	Overall survival and time to next treatment was assessed at the University hospitals in Heidelberg and Barcelona.

Plants

Seed stocks

Report on the source of all seed stocks or other plant material used. If applicable, state the seed stock centre and catalogue number. If plant specimens were collected from the field, describe the collection location, date and sampling procedures.

Novel plant genotypes

Describe the methods by which all novel plant genotypes were produced. This includes those generated by transgenic approaches, gene editing, chemical/radiation-based mutagenesis and hybridization. For transgenic lines, describe the transformation method, the number of independent lines analyzed and the generation upon which experiments were performed. For gene-edited lines, describe the editor used, the endogenous sequence targeted for editing, the targeting guide RNA sequence (if applicable) and how the editor was applied.

Authentication

Describe any authentication procedures for each seed stock used or novel genotype generated. Describe any experiments used to assess the effect of a mutation and, where applicable, how potential secondary effects (e.g. second site T-DNA insertions, mosiacism, off-target gene editing) were examined.

Flow Cytometry

Plots

Confirm that:

- [x] The axis labels state the marker and fluorochrome used (e.g. CD4-FITC).
- [x] The axis scales are clearly visible. Include numbers along axes only for bottom left plot of group (a 'group' is an analysis of identical markers).
- [x] All plots are contour plots with outliers or pseudocolor plots.
- [x] A numerical value for number of cells or percentage (with statistics) is provided.

Methodology

Sample preparation	Described in the Methods section
Instrument	Provided in the Methods section and Suppl. Data 2
Software	Provided in the Methods section and Suppl. Data 2
Cell population abundance	Provided in the Methods section
Gating strategy	Gating strategies are provided in Suppl. Figure 5.

- [x] Tick this box to confirm that a figure exemplifying the gating strategy is provided in the Supplementary Information.
